# Characterizing gastrointestinal dysfunction after pancreatic resection: a single-center retrospective study

**DOI:** 10.1186/s12876-022-02565-7

**Published:** 2022-11-26

**Authors:** Rebecca Bromley-Dulfano, Auriel T. August, Amy Y. Li, Walter Park, Brendan Visser

**Affiliations:** 1 Stanford University School of Medicine, 291 Campus Drive, Stanford, CA 94305 USA; 2 Department of General Surgery, Stanford Hospital, Stanford, CA USA; 3 Department of Medicine, Division of Gastroenterology & Hepatology, Stanford Hospital, Stanford, CA USA

**Keywords:** Pancreaticoduodenectomy, Whipple, Exocrine pancreatic insufficiency, EPI, Small intestinal bacterial overgrowth, SIBO, Delayed gastric emptying, DGE, Gastrointestinal dysfunction, Gastrointestinal symptoms, Pancreatic resection

## Abstract

**Background:**

There are many well-described potential gastrointestinal (GI) side effects of pancreatic resection that can cause patients to suffer from chronic malabsorption, diarrhea, and persistent nausea. These GI symptoms can affect postoperative recovery, initiation of adjuvant therapy, and overall quality of life (QOL). The purpose of this study is to quantify the incidence of post-procedural complications and identify patients at higher risk for experiencing GI dysfunction after pancreatectomy.

**Methods:**

A retrospective review of patients who underwent pancreatic resection at a single institution between January 2014 and December 2019 was performed. Demographics, operative factors, and postoperative gastrointestinal symptomatology and treatments were obtained by chart review. Significance tests were performed to compare GI dysfunction between patient subgroups.

**Results:**

A total of 545 patients underwent pancreatic resection; within the cohort 451 patients (83%) underwent a pancreaticoduodenectomy (PD) and the most common indication was pancreatic adenocarcinoma. Two-thirds of patients (67%) reported gastrointestinal symptoms persisting beyond hospitalization. Only 105 patients (20%) were referred to gastroenterology for evaluation with 30 patients (5.5%) receiving a formal diagnosis. Patients who underwent PD were more likely to report GI symptoms and patients who identified as Caucasian were more likely to be referred to gastroenterology for evaluation.

**Conclusions:**

Gastrointestinal dysfunction after pancreatic resection occurs frequently yet only a small percentage of patients are referred for formal testing and diagnosis. There also appears to be a racial difference in referral patterns. Patients would benefit if earlier attention was dedicated to the diagnosis and corresponding treatment for postoperative digestive health disorders to optimize treatment planning and QOL.

## Introduction

Pancreatic resections are performed for various pathologies; from relatively benign cystic lesions to highly malignant adenocarcinomas. Although pancreatic cancer is a grave diagnosis with a 5-year survival rate of only 34% for localized disease [1], patients who undergo pancreatic resection for a cystadenoma will likely live a normal lifespan beyond surgery. Complications after pancreatic resection, such as leaks and fistulas have established diagnostic criteria and treatment measures. What remains a more elusive task is diagnosing and treating the gastrointestinal (GI) dysfunction that arises after pancreatic resection. As surgical treatment and postoperative management for pancreatic pathology have advanced over time, it is necessary to focus on improving patient quality of life (QOL) after resection. While often dismissed, GI dysfunction can affect a patient’s clinical course, time to adjuvant therapy, and overall post-operative quality of life.

In a single-institution chart review study consisting of 52 patients undergoing pancreatic resection, 50% of patients reported new or different GI symptoms (as a metric for dysfunction) post-operatively, with some symptoms persisting over two years after surgery [2]. While GI symptoms are expected in the immediate post-operative period, studies like this show that symptoms can persist well beyond the first few weeks after surgery. A similar study performed for patients who underwent surgery for gastric or esophageal cancer found that there was a 73% prevalence of malabsorption and malnutrition due to gastrointestinal changes, even 18 months after surgery [3].

Little work has been done to truly validate how often GI dysfunction arises in the postoperative period and whether these syndromes are being diagnosed and treated adequately. The presentation of symptoms is relevant at all time points, as early symptom presentation can delay the critical initiation of adjuvant chemotherapy, while delayed presentation can severely impact patients' quality of life. The purpose of this study is to determine the incidence of gastrointestinal dysfunction arising after pancreatic resection and determine whether patients are formally diagnosed and treated. Further, we want to determine what risk factors should urge a surgeon to consider an earlier referral to GI for evaluation and management.

## Methods

A retrospective review of patients who underwent pancreatic resection at a single institution between January 2014 and December 2019 was performed. Stanford University Institutional Review Board Committee approval was obtained for IRB Protocol 48933 on March 4, 2019. Demographics, operative details, and postoperative gastrointestinal symptomatology and treatments were obtained by chart review. Gastrointestinal symptoms included: nausea/vomiting, diarrhea/steatorrhea, poor oral tolerance, pain/bloating, constipation, and reflux. Additional documentation including referral to gastroenterology for persistent symptoms, further testing and evaluation, and formal diagnosis for gastrointestinal dysfunction were also obtained. The presence of GI symptoms was assessed from outpatient clinic documentation filed after the initial hospitalization. Patients without any documented follow-up after their initial hospitalization were excluded from the study. Significance tests were performed to compare GI dysfunction between patient groups depending on demographics, diagnoses, and operation characteristics. Non-normally distributed continuous data were reported as (median [Interquartile Range]). Normally distributed data were reported as (mean [standard deviation]). Continuous normally distributed variables were compared using ANOVA, while continuous non-normal variables were compared using the Wilcoxon rank-sum (2 groups) or Kruskal-Wallis (>2 groups) test. Categorical and binary variables were compared using Pearson's chi-squared. Descriptive analyses were conducted using STATA 14 (ver. 2, College Station, TX).

## Results

During the five-year study period, a total of 560 patients underwent a pancreatic resection. Fifteen patients did not have follow-up documentation and were excluded from the analysis, thus a total of 545 patients were included in the final cohort. Within this cohort, 50% were male (*n*=274), the median age was 66 [57-74] and 57% were white (*n*=313) (Table 1). The most common diagnoses were adenocarcinoma (*n*=333, 61%), followed by neuroendocrine tumor (*n*=58, 11%), intraductal papillary mucinous neoplasm (IPMN)/intraductal tubulopapillary neoplasm (ITPN) (*n*=56, 10%), solid pseudopapillary tumors (*n*=29, 5.3%), benign tumors (other cysts and pancreatitis) (*n*=36, 6.6%), and other carcinomas including metastatic disease (*n*=33, 6.1%). The operations performed included classic pancreaticoduodenectomy (CPD) (56.2%), pylorus preserving pancreaticoduodenectomy (PPPD) (26.6%), distal pancreatectomy (13.2%), total or completion pancreatectomy (3.1%) and other operations (0.9%), such as distal pancreatectomy with celiac access resection (Table 2). The median length of stay (LOS) was 9 [7-13] days for the entire cohort.

**Table 1:** Patient demographics by gender, race, and age. The data are presented as the total number of patients (n) and their percentage out of the total study cohort, *n*=545 patients. Age is presented as age group buckets and median age is reported given the data were non-normally distributed

Category	Demographics	Patients, n (%)
**Gender**	Male	274 (50.3)
	Female	271 (49.7)
**Race**	White, Non-Hispanic	313 (57.4)
	Asian and Pacific Islander, Non-Hispanic	111 (20.4)
	Black, Non-Hispanic	7 (1.3)
	Hispanic	87 (16.0)
	Other, Non-Hispanic	27 (5.0)
**Age**	<60 yo	168 (30.8)
	60-74 yo	257 (47.2)
	75+ yo	120 (22.0)
	Median (IQR)	66 (57-74)

**Table 2:** Breakdown of Study Cohort by Type of Surgical Procedure Performed. Data in the left column are presented as the total number of patients (n) and their percentage out of *N*=545 patients. The right column reports the number and percent of symptomatic patients among those who received each respective type of procedure (denominator is the of patients from left column)

Surgery Performed	Patients, n (%)	Symptomatic Patients, n(%)
Classic Pancreaticoduodenectomy	306 (56.2)	215 (70.3)
Pylorus-Sparing Pancreaticoduodenectomy	145 (26.6)	100 (69.0)
Distal pancreatectomy	72 (13.2)	72 (47.2)
Total pancreatectomy	17 (3.1)	15 (88.2)
Other (e.g., Appleby)	5 (0.9)	2 (40)

**Fig. 1:**
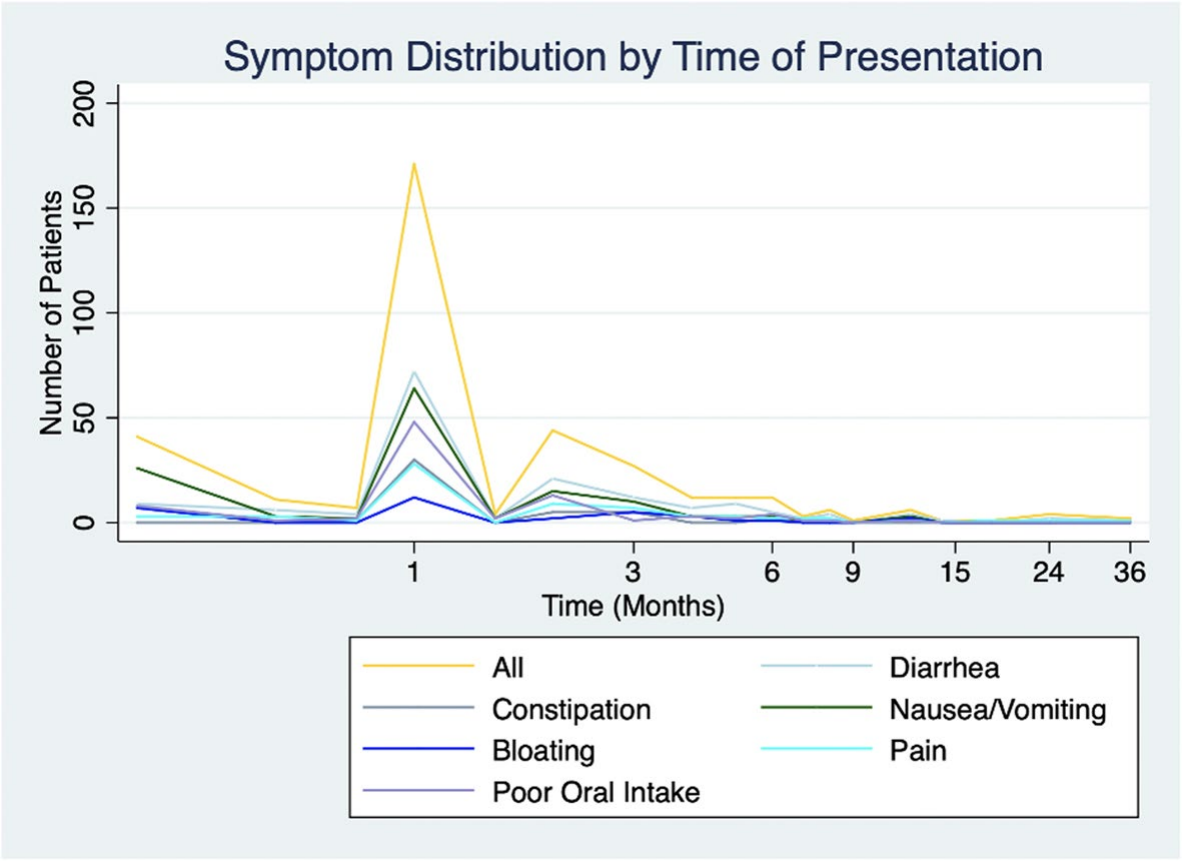
Symptom Distribution by Time of Presentation. Shown above is the number of patients with select GI symptoms plotted according to the time of symptom presentation (in months)

Two-thirds of all patients (*n*=366, 67%) reported gastrointestinal symptoms persisting beyond their initial hospitalization. Most of the symptomatic cohort (*n*=277, 76%) first reported GI symptoms within the first three months after surgery with the peak incidence at four weeks post-operatively (Fig. 1). The remaining symptomatic patients (*n*=89, 24%) first reported symptoms beyond three months after surgery (Fig. 1). Diarrhea (*n*=161, 30%) was the most reported symptom, followed by nausea or vomiting (*n*=136, 25%) and poor oral intake or weight loss (*n*=87, 16%) (Table 3). Patients who underwent PD (either classic or pylorus-preserving) were more likely to experience GI dysfunction compared with patients who underwent all other procedures (69.8% vs 54.3% respectively, *p*=0.003). Patients who underwent a total pancreatectomy did not show a significant difference in their likelihood of experiencing GI dysfunction compared with those who underwent PD (88.2% vs 69.8%, *p*=0.10), and patients who underwent distal pancreatectomy were less likely to experience GI dysfunction compared with those who underwent PD (47.2% vs 69.8%, p<0.001). There was no difference in symptoms between the group of patients who underwent CPD versus PPPD (68% vs 70%, *p*=0.78). Whether patients experienced GI dysfunction or not, did not vary significantly by age, gender, or race. Roughly half of symptomatic patients (*n*=176, 48%) required Pancreatic Enzyme Replacement Therapy (PERT) following surgery, the majority of whom (*n*=148, 85% of patients taking PERT) were initiating PERT for the first time.

**Table 3:** Number and Percentage of GI Symptoms Among Symptomatic Patients. Data are presented as the total number of symptomatic patients (n) and the percentage they represent (%), note the denominator of the percentage is the total number of symptomatic patients (*N*=365) and not the entire study cohort

Symptom Reported	Symptomatic Patients *N*=365, n (%)
Diarrhea/steatorrhea	161 (29.5)
Nausea/Vomiting	136 (24.9)
Pain/bloating	100 (18.3)
Poor Oral Intake/Weight loss	87 (16.0)
Constipation	44 (8.1)
Reflux	10 (1.8)

As in Table 4, only 105 patients (19% of all patients, 27% of symptomatic patients) were referred to gastroenterology for further evaluation. Symptomatic patients were most commonly treated empirically with enzyme replacement therapy (*n*=148, 40%), proton-pump inhibitors (*n*=132, 36%), cathartic medications (*n*=115, 31%), and antiemetics (*n*=90, 25%). The most common indications for referral were diarrhea (46%), nausea and vomiting (38%), and pain (26%). The likelihood of placing a referral varied significantly by race, with 33% of all symptomatic non-Hispanic white patients (*n*=70 of 213) receiving referrals, compared with only 17% of Asian and Pacific Islander patients (*n*=13 of 75), 16% of Hispanic patients (*n*=9 of 55), and 0% of Black patients (*n*=0 of 4). Patients identifying as non-Hispanic white were significantly more likely to receive a referral (32.9%) than all other races/ethnicities (19%, *p*=0.003). There was no significant difference in referral rates between males (30%) and females (25%, *p*=0.29), nor among age groups (<60 years old (31%), 60-70 years old (25%), or >70 years old (26%, *p*=0.45)). Referral rates also did not vary by diagnosis (*p*=0.56) or by operation type (*p*=0.12). Among symptomatic patients, only 3% (*n*=12) received a nutrition referral, all of which occurred at or after the time of symptom presentation (Table 4).

**Table 4:** Trends in treatment type, referral rates, and PERT usage changes pre- and post-operatively. Data are presented as the total number of symptomatic patients (n) and the percentage they represent (%), note the denominator of the percentage is the total number of symptomatic patients (*N*=36) and not the entire study cohort

Treatment	Symptomatic patients receiving treatment (%), *n*=365
PERT	176 (48%)
Proton Pump Inhibitor	132 (36%)
Cathartic	115 (31%)
Anti-emetic	90 (25%)
Appetite Stimulant	12 (3%)
Antibiotic	6 (1.5%)
**Referral Rates**	
GI Referral	105 (29%)
Nutrition Referral	12 (3%)
**PERT Usage among Symptomatic Patients**	
Never used PERT	182 (50%)
Initiated PERT following Surgery	148 (40%)
Previously used PERT; continued Post-Op	28 (8%)
Pre-op PERT; no Post-op PERT	8 (2%)

All 105 patients referred to gastroenterology underwent some form of testing for GI dysfunction. This included stool elastase (*n*=16, 3%), glucose breath test (*n*=8, 2.2%), esophagogastroduodenoscopy (EGD, *n*=32, 8.7%), Colonoscopy (*n*=8, 2.2%) or gastric emptying study (*n*=15, 4.1%). Of the patients who underwent testing, 22 patients (4% of total study cohort) were formally diagnosed with exocrine pancreatic insufficiency (EPI) by a positive stool elastase test, 11 patients (2%) with delayed gastric emptying (DGE) on upper GI study, and 7 patients (1.2%) with small intestinal bacterial overgrowth (SIBO) by a positive glucose breath test.

## Discussion

Gastrointestinal dysfunction after pancreatic resection is a known potential complication, yet it remains underdiagnosed and undertreated. In this study, despite postoperative gastrointestinal symptoms occurring in 67% of the cohort, only 19% of patients were referred to gastroenterology for formal testing and diagnosis. Specifically, patients who undergo PD are at the greatest risk for developing prolonged GI symptoms and could benefit from aggressive early management of their symptoms in order to avoid delaying adjuvant therapy and improve QOL. Previous studies have documented various types of gastrointestinal dysfunction as one of the complications after pancreatectomy, however, these studies are limited in sample size and vary in terms of the methods of quantifying the dysfunction.

In this large retrospective cohort study, we found that patients who undergo PD (either classic or pylorus-preserving) were more likely to experience GI dysfunction (70%) as compared to patients who had other procedures such as total or distal pancreatectomies (54%, *p*=0.003). By contrast, patients who underwent a distal pancreatectomy were less likely to experience GI dysfunction after surgery (47.2%) compared with patients who underwent other procedures (70%, *p*<0.001). Surprisingly, there was no significant difference in symptom presentation between patients undergoing CPD versus PPPD. Patients with pancreatic adenocarcinoma are more likely to spend longer with the disease in situ resulting in greater preoperative pancreatic atrophy and greater impaired postoperative pancreatic function. It is surprising, however, that patients who underwent total pancreatectomy did not have a higher incidence of symptoms given that any pancreatic tissue is absent post-operatively. This could be attributed to greater attention and aggressive prophylactic management of the expected changes in GI function for these patients.

Another important outcome of this study is the timing of symptom reporting by patients. Based on the current study, there was the greatest incidence of symptoms reported around 4–6 weeks post-operatively. This is significant as most patients who undergo resection for adenocarcinoma this is the window in which they should begin adjuvant chemotherapy. Because of this, GI symptoms reported during the first six weeks risked delaying initiating chemotherapy in these patients. Similarly, there were a number of patients who reported symptoms beyond six months after surgery. Although symptoms that arise during this later timeline may not delay treatment, they still can still impact the patient’s clinical course and quality of life. The symptoms of EPI are highly correlated with poor nutritional status and impairment in quality of life [4, 5]. A systematic review of EPI after pancreaticoduodenectomy found an incidence of 38-93% when confirmatory testing was performed [6]. The authors demonstrated that most patients who reported symptoms of diarrhea/steatorrhea, bloating or poor weight gain were started on PERT, yet only 4% of patients went on to undergo fecal elastase testing to confirm a diagnosis of EPI, similar to the incidence in our study of patients who underwent formal testing. These findings suggest EPI is underreported, underdiagnosed, and/or misdiagnosed. For this reason, earlier testing and intervention is needed in these patients. We recommend closer monitoring for symptoms beginning 4-6 weeks after surgery and earlier consideration for referral to GI specialists (and to other services such as nutrition) as patients recover. Our findings also imply a larger general trend of under-referral. One factor that may underlie these referral patterns could be that gastroenterologists and nutritionists may have less experience with managing patients after pancreatic surgeries, which may in turn create hesitancy on the part of operating surgeons in referral.

DGE is one of the most common and easily recognized complications after pancreatic resection occurring in about 12-14% of patients [7]. Most are identified and treated during the index hospitalization given the tell-tale symptoms of nausea and vomiting. One study found that 25% of patients reported symptoms of nausea/vomiting extending beyond their hospitalization and 2% of the study’s cohort were diagnosed with DGE via upper GI fluoroscopy [8]. Our study found similar results, with 2% of our cohort being diagnosed with DGE. While this is only a small percentage of patients, it is nonetheless important to consider this diagnosis outside of the hospital.

Our results show there is a persistent underdiagnosis and undertreatment of GI dysfunction after pancreatic resection. There were differential referral rates for patients across different races/ethnicities. Non-white identifying patients with post-operative symptoms were less likely to receive a referral and notably, none of the Black-identifying patients with symptoms received a referral. While the sample size of Black patients with GI dysfunction was small (*n*=4), this trend is concerning for inequity between treatments. There of course may be various factors at play, from the difference in patient self-advocacy to the impact of clinician implicit bias. Interventions could be implemented to ensure that patients of color are not being missed or overlooked for referrals to specialty care such as: standardizing referral decision support tools, adding referral flags in patient charts, and/or increasing clinician awareness around this disparity.

This study is limited by its retrospective nature. There is no standardized documentation of GI symptoms before and/or after pancreatic resection at our institution, making it difficult to fully ascertain the extent and severity of symptoms. In addition, this dataset focuses specifically on the constellation of symptomatology, onset and timing of symptoms, and referral and treatments, but does not currently include the impact of adjuvant chemotherapy or surgical complications on GI dysfunction. However, a strength of this study is the large sample size and long study period over five years. Future directions for this research include a prospective study attempting to monitor a patient's gastrointestinal health post-operatively and intervening with GI referral, if warranted, to further elucidate the impact of these variables, as well as to evaluate how adjuvant chemotherapy and how surgical complications can impact postoperative GI dysfunction.

## Conclusion

Gastrointestinal dysfunction after pancreatectomy remains underdiagnosed and undertreated. Patients with untreated GI symptoms and underlying dysfunction after pancreatic surgery can end up with delayed chemotherapy and poor quality of life. The results of this study may help better identify patients who are at higher risk for developing GI dysfunction and to improve their follow up treatment, and clinical course.

## Data Availability

The datasets used and/or analyzed during the current study are available from the corresponding author on reasonable request.

## Abbreviations

CPD: Classic Pancreaticoduodenectomy
DGE: Delayed gastric emptying
EGD: Esophagogastroduodenoscopy
EPI: Exocrine pancreatic insufficiency
GI: Gastrointestinal
QOL: Quality of life
IPMN: Intraductal papillary mucinous neoplasm
ITPN: Intraductal tubulopapillary neoplasm
PD: Pancreaticoduodenectomy
PPPD: pylorus preserving pancreaticoduodenectomy
SIBO: small intestinal bacterial overgrowth
